# Does cervical facet degeneration impact surgical outcomes and sagittal balance in patients with radiculopathy?

**DOI:** 10.1186/s12893-021-01227-x

**Published:** 2021-04-30

**Authors:** Zheng Wang, Zhen Liu, Zhi-Wei Wang, Wen-Yuan Ding, Da-Long Yang

**Affiliations:** Department of Spinal Surgery, The Third Hospital of Hebei Medical University, 139 Ziqiang Road, Shijiazhuang, 050051 People’s Republic of China

**Keywords:** Facet degeneration, Radiculopathy, Anterior cervical discectomy and fusion, Sagittal balance, Surgical outcomes

## Abstract

**Purpose:**

In our present study, we aimed to investigate (1) whether cervical facet degeneration (FD) affects the clinical functional scores of patients with cervical radiculopathy after single-segment anterior cervical discectomy fusion (ACDF) and (2) whether FD affects the sagittal parameters of the cervical spine.

**Methods:**

A total of 120 enrolled patients who underwent single-segment ACDF for radiculopathy with more than 2 years of follow-up were classified into two groups based on whether the preoperative mean FD was greater than or less than the mean FD grading score: mild FD group (mean score ≤ 2, n = 102) or severe FD group (mean score > 2, n = 48). Sagittal alignment changes and clinical functional scores were compared between the 2 groups. The relevant factors for FD were identified using multivariate logistic regression.

**Results:**

Age, duration of symptoms, disc height and interfacet distance were independently associated with preoperative FD (age: *P* < 0.001; duration of symptoms: *P* = 0.020; disc height: *P* < 0.001; interfacet distance: *P* = 0.045). Compared with the mild FD group, the preoperative VAS (neck pain) score and NDI of the severe FD group were also higher, and the improvement of neck symptoms was better during the follow-up period. However, all clinical scores and radiographic parameters showed no significant differences during the 2-year follow-up. Additionally, no significant differences in the sagittal parameter changes were presented.

**Conclusion:**

Patients with severe FD tended to experience more severe neck pain before surgery and greater improvement of neck symptoms at the follow-up visit. However, 2-year clinical efficacy and sagittal alignment after ACDF may not be markedly affected by preoperative FD severity. ACDF is considered to be a good choice for patients with radiculopathy, especially for patients with severe FD.

## Introduction

Anterior cervical discectomy fusion (ACDF) has been regarded as a common surgical procedure for treating cervical degenerative diseases [1] and is related to kyphosis correction and the maintenance of postoperative lordosis [2, 3]. ACDF is considered to be an established procedure for the treatment of neck pain and radiculopathy. Evidence suggests that neck axial pain caused by facet degeneration (FD) accounts for greater than half of all cervical pain [4]. In addition, the morbidity of facet joint pain in patients with chronic neck pain is as high as 60% [5]. The facet joints of the cervical spine were also crucial in guiding the movement of the cervical spine and distributing the axial load. However, narrow space, subchondral bone erosion, and osteophyte hypertrophy will gradually occur due to the influence of degeneration [6, 7]. Severe FD can also cause myelopathy and degenerative cervical spondylolisthesis [8, 9]. Patients with severe FD are commonly encountered during ACDF in the clinic. Previous studies have shown that the preoperative severity of FD does not impact the clinical outcomes and sagittal balance of patients with cervical spondylotic myelopathy after laminoplasty [10], but whether preoperative FD degeneration affects the postoperative sagittal balance and clinical results in patients with cervical radiculopathy receiving ACDF remains unknown. Compared with laminoplasty, ACDF requires the insertion of a large cage, which may change the tension of the facet joints, thereby pulling the narrow joint capsule. In addition, sagittal alignment is crucial to cervical function. Sagittal parameter analysis is critical to understanding cervical balance and predicting clinical outcomes. According to previous literature [11, 12], the quality of life of patients may be affected by sagittal parameter changes. Some cervical sagittal parameters are potentially associated with the scores of postoperative functions [3, 13]. The relationship between cervical sagittal parameters and FD has not yet been reported.

Therefore, this article aims to assess the effect of FD on the clinical functional scores and sagittal parameters of patients with cervical radiculopathy after single-segment ACDF. Our conclusions can help surgeons predict postoperative outcomes and develop more effective surgical strategies.

## Methods

### Patient population

The study was approved by the ethical committee of Third Affiliated Hospital of Hebei Medical University. All the patients were given informed consent and all methods were carried out in accordance with relevant guidelines and regulations. Cervical spondylotic radiculopathy patients admitted to our hospital receiving single-segment ACDF from January 2015 to December 2017 were retrospectively reviewed. Inclusion criteria: (1) cervical spondylotic radiculopathy (single segment) patients diagnosed according to the clinical manifestations and radiological analyses; (2) patients whose symptoms did not improve after conservative treatment; (3) patients whose cervical imaging were clear and could be accurately measured; (4) patients' follow-up were not less than 2 years and the follow-up data were complete. Exclusion criteria included the following: (1) history of previous cervical surgery; (2) combined with trauma, tumour, or infection; (3) ankylosing spondylitis and rheumatoid arthritis; and (4) imaging data could not be measured before and after surgery. Imaging studies included plain radiography, computed tomography (CT) and magnetic resonance imaging (MRI) of the cervical spine. We assessed clinical outcomes preoperatively and during the follow-up period, including the Neck Disability Index (NDI) and visual analogue scale (VAS) scores for arm and neck pain individually and the Short Form-36 (SF-36).

### Radiographic analysis

**Fig. 1:**
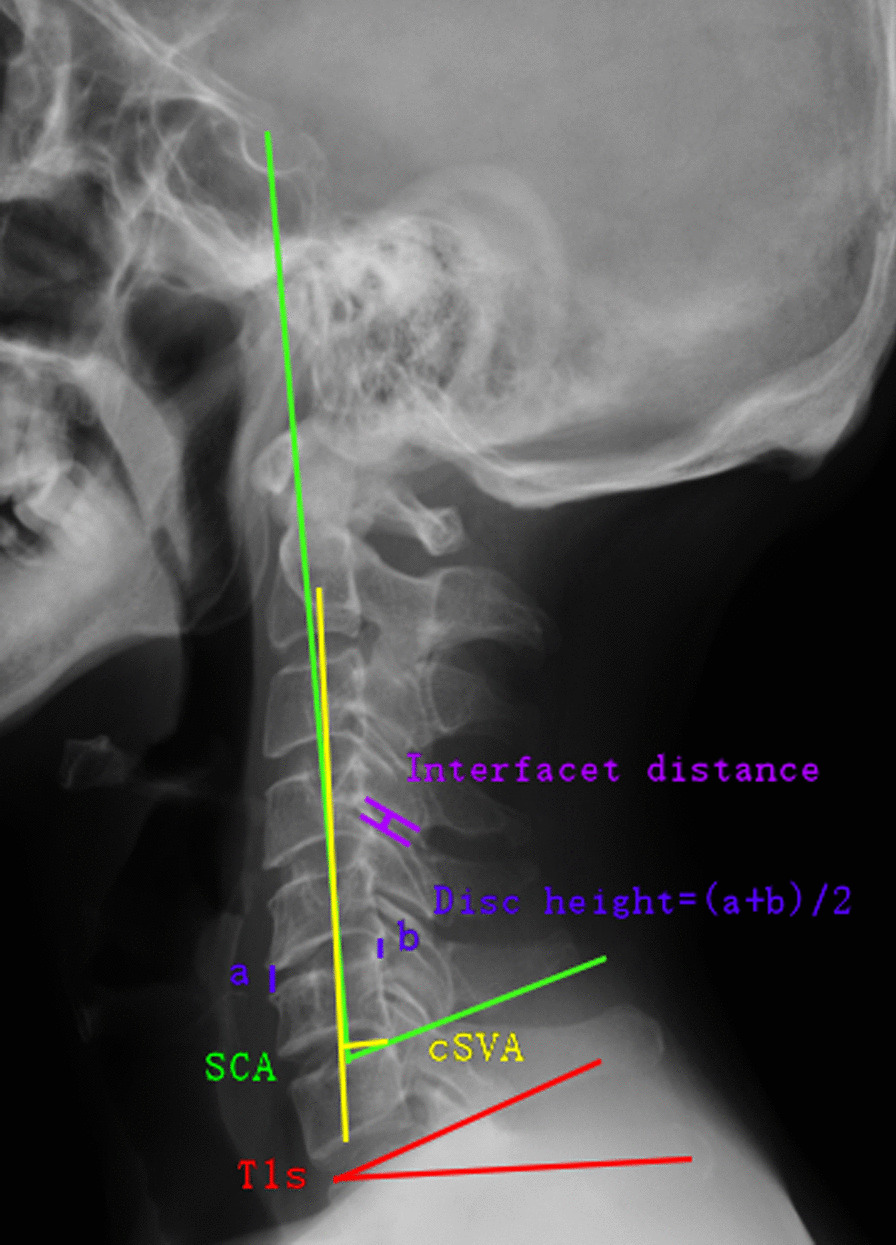
SCA is considered as the angle between the C7 slope and the straight line connecting the middle of the Sella turcica and the middle of the C7 endplate. cSVA is regarded as the distance between the C7 posterior superior corner and the plumbline from the center of C2 body. T1s is defined as the angle between the T1 superior endplate and a horizontal line. Disc height is calculated as the mean value of the anterior (**a**) and posterior (**b**) disc height. Interfacet distance is regarded as the interfacet distance at the most severely degenerated level

**Fig. 2:**
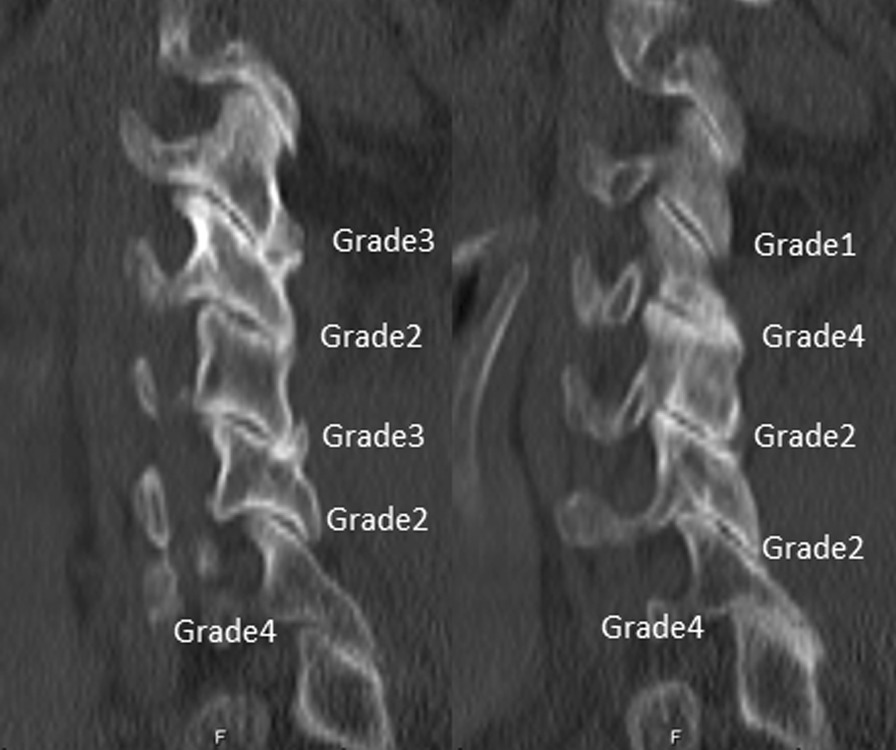
Grade1: normal; Grade2: degenerative changes, including joint space narrowing, cyst formation, and small osteophytes without joint hypertrophy; Grade3: joint hypertrophy secondary to large osteophytes without fusion; Grade4: bony fusion of the joint

Sagittal balance parameters, such as spino cranial angle (SCA), sagittal vertical axis of C2–C7 (cSVA), and T1-slope (T1s), obtained from plane-neutral lateral radiographs were measured preoperatively and at follow-up. The SCA is considered the angle between the C7 slope and the straight line connecting the middle of the sella turcica and the middle of the C7 endplate. cSVA is regarded as the distance between the C7 posterior superior corner and the plumbline from the centre of the C2 body. T1s is defined as the angle between the T1 superior endplate and a horizontal line. Disc height was calculated as the mean value of the anterior (a) and posterior (b) disc height. The interfacet distance is regarded as the interfacet distance at the most severely degenerated level (Fig. 1). The degree of FD was classified into 4 types (scores) based on the preoperative CT sagittal images (Fig. 2). We evaluated the severity of FD in all segments of each patient and calculated the average value from C2/3 to C7/T1. Patients enrolled were then categorized into the mild FD group and severe FD group based on the average value of FD scores, and the cut-off value between the two groups was defined as the mean value of the average FD score.

### Statistical analysis

Data are revealed as the median and interquartile ranges or mean ± standard deviation in each group. SPSS software (version 22.0; SPSS Inc., Chicago, IL, USA) was applied to calculate and analyse the data in the study. The independent-sample t-test, χ^2^ test, and Mann–Whitney U test were applied for comparison of independent variables between two groups. Relevant factors of preoperative facet degeneration in cervical spondylotic radiculopathy patients with ACDF were analysed using multivariate logistic regression analysis, and the results are presented as adjusted odds ratios (aORs) and 95% confidence intervals (CIs). Significance was noted at the *P* < 0.05 level.

## Results

### The impact of FD score on the patient backgrounds

The degree of FD was classified into 4 types (scores) in Table 1. A total of 120 enrolled patients in the current study were classified into two groups based on whether the preoperative mean FD was greater than or less than the mean grading score: mild FD group (≤ 2, n = 102) or severe FD group (> 2, n = 48). Patient clinical features according to preoperative FD are presented in Table 2. Age (44.7 vs 51.4 years, *P* < 0.001) and the duration of symptoms (10.06 vs 13.88 months, *P* < 0.001) in the severe FD group were remarkably higher than those in the mild FD group. In contrast, the disc height (4.06 vs 3.79 mm, *P* < 0.001) and interfacet distance (2.44 vs 1.96 mm, *P* < 0.001) in the severe FD group were markedly lower than those in the mild FD group. However, no statistically significant difference in sex (*P* = 0.591) or incidence of Modic change (*P* = 0.882) was noted between the FD groups.

**Table 1:** Facet degeneration grading

Grade1	Normal
Grade2	Degenerative changes, including joint space narrowing, cyst formation, and small osteophytes without joint hypertrophy
Grade3	Joint hypertrophy secondary to large osteophytes without fusion
Grade4	Bony fusion of the joint

**Table 2:** Comparison of patient backgrounds according to preoperative facet degeneration

	Mild-FD group	Severe-FD group	*p*-value
No. of patients	102	48	
Mean grading score of FD	≤ 2	> 2	
Age (year)	44.70 ± 5.22	51.40 ± 4.66	< 0.001
Sex (male/female)	60/42	26/22	0.591
Duration of symptoms (months)	10.06 ± 4.63	13.88 ± 5.11	< 0.001
Incidence of Modic Change	18(17.6%)	8(16.7%)	0.882
Disc height (mm)	4.06 ± 0.24	3.79 ± 0.30	< 0.001
Interfacet distance (mm)	2.44 ± 0.62	1.96 ± 0.54	< 0.001

FD, facet degeneration

### The impact of FD score on the balance parameter changes

No significant difference among cervical sagittal parameters, including SCA, cSVA and T1s, before the operation or at the follow-up period existed between the 2 groups. Additionally, no marked differences in the above sagittal parameter changes between the 2 groups were presented (△SCA, *P* = 0.067; △cSVA, *P* = 0.395 and △T1s, *P* = 0.810, respectively). However, compared with preoperative values, all radiographic parameters except T1s in both FD groups showed remarkable differences (severe FD: SCA, *P* < 0.001; cSVA, *P* < 0.001; T1s, *P* = 0.094. mild FD: SCA, *P* < 0.001; cSVA, *P* < 0.001; T1s, *P* = 0.173) (Table 3).

**Table 3:** Comparison of radiologic and clinical parameters according to preoperative facet degeneration

	Mild-FD group	Severe-FD group	*p*-value
T1s (°)			
Pre	25.39 ± 5.30	25.86 ± 6.30	0.634
F/U	25.80 ± 5.61	26.67 ± 6.35	0.401
△T1s (°)	0.46 ± 3.00	0.81 ± 3.25	0.516
Pre vs F/U	0.173	0.094	
cSVA (mm)			
Pre	18.19 ± 6.19	17.77 ± 7.19	0.718
F/U	23.44 ± 6.96	21.94 ± 7.89	0.237
△cSVA (mm)	5.26 ± 6.05	4.16 ± 6.23	0.307
Pre vs F/U	< 0.001	< 0.001	
SCA (°)			
Pre	82.21 ± 10.63	81.05 ± 5.81	0.298
F/U	78.38 ± 9.36	78.27 ± 7.16	0.798
△SCA (°)	− 3.83 ± 5.30	− 2.78 ± 4.08	0.067
Pre vs F/U	< 0.001	< 0.001	

Pre, preoperative; F/U, follow up; FD, facet degeneration

T1s, T1-Slope, cSVA, C2–7 sagittal vertical axis, SCA , spino cranial angle

### The impact of FD score on the analysis of clinical outcomes

**Fig. 3:**
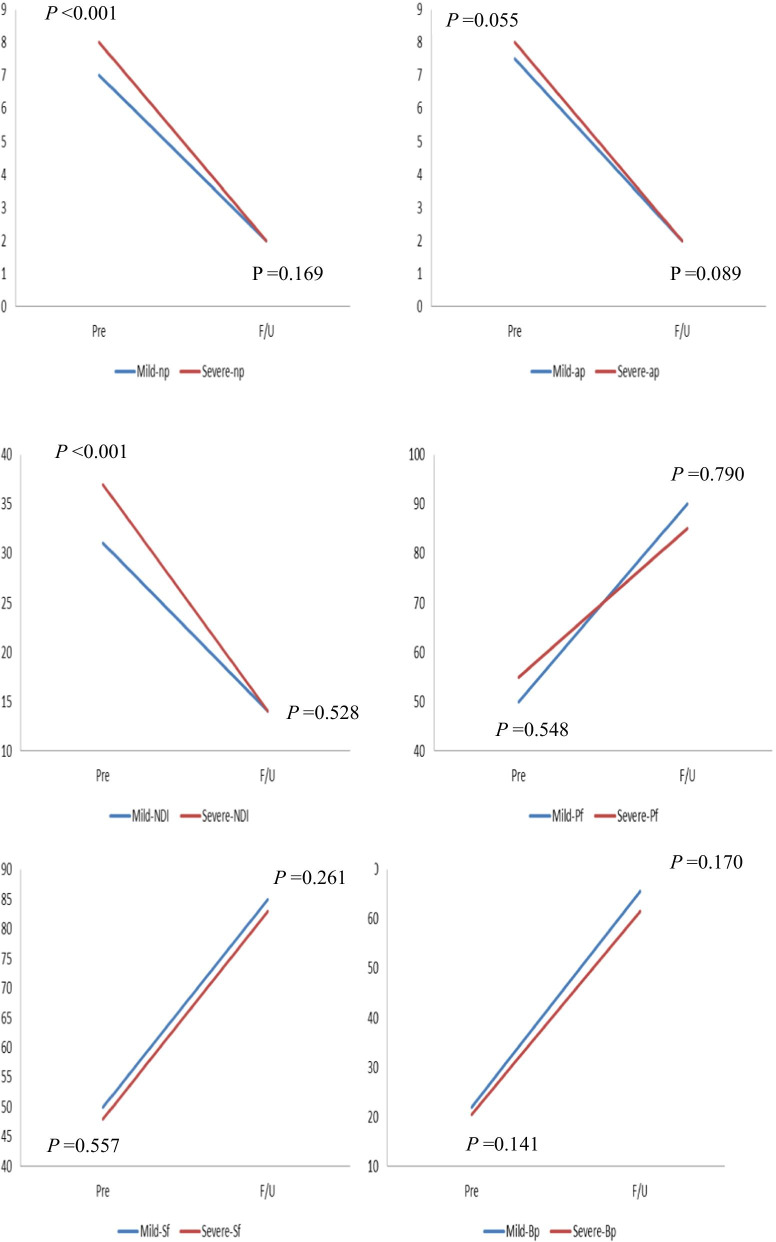
The changes of clinical scores in both 2 groups

**Fig. 4:**
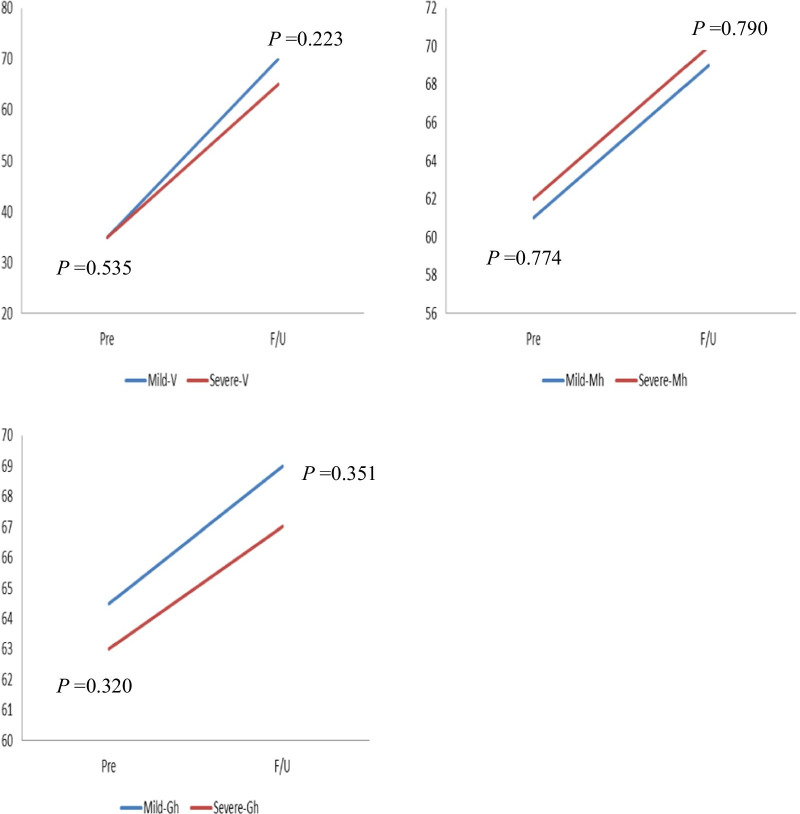
The changes of clinical scores in both 2 groups

Before treatment, the patients in the severe FD group had similar scores to those in the mild FD group for all measures except VAS (neck pain) and NDI (neck pain: *P* < 0.001; NDI: *P* < 0.001). Scores for neck pain and NDI in the severe FD group were significantly greater in the mild FD group with radiculopathy preoperatively. However, during the follow-up period, none of the clinical indicators, including VAS, NDI and SF-36, showed significant differences. During the follow-up period, both VAS of neck pain and arm pain and NDI showed significant improvement in both groups. For the SF-36, compared to the preoperative values, most indicators, including social functioning, bodily pain, physical functioning, mental health, and vitality, were significantly enhanced, except for general health in both FD groups (Table 4). However, we found that the mean ± standard deviation values of △VAS (neck pain) and △NDI (changes at 2-year follow-up compared to preoperative period) were 4.52 ± 1.68 and 17.44 ± 5.13 in the mild FD group and 5.23 ± 1.21 and 21.48 ± 4.83 in the severe FD group, respectively. These differences were significant (△VAS (neck pain): *P* = 0.010, △NDI: *P* < 0.001). No marked differences in other clinical outcome changes between the two FD groups were found (Table 5). In addition, the line chart also intuitively showed the changes in clinical scores in both groups (Figs. 3 and 4).

**Table 4:** Comparison of clinical outcomes according to preoperative facet degeneration

		Mild-FD group	Severe-FD group	*p*-value
VAS				
Neck pain				
Pre	Median	7	8	< 0.001
	IQR	5–8	7–8	
F/U	Median	2	2	0.169
	IQR	2–3	2–3	
Pre vs F/U		< 0.001	< 0.001	
Arm pain				
Pre	Median	7.5	8	0.055
	IQR	7–8	7–8	
F/U	Median	2	2	0.089
	IQR	1–3	1–3	
Pre vs F/U		< 0.001	< 0.001	
NDI				
Pre	Median	31	37	< 0.001
	IQR	27–36	32–39	
F/U	Median	14	14	0.528
	IQR	11–17	12–17	
Pre vs F/U		< 0.001	< 0.001	
SF-36				
Physical functioning				
Pre	Median	50	55	0.548
	IQR	45–65	50–65	
F/U	Median	90	85	0.980
	IQR	80–95	80–90	
Pre vs F/U		< 0.001	< 0.001	
Social functioning				
Pre	Median	50	48	0.557
	IQR	43–55	43–53	
F/U	Median	85	83	0.261
	IQR	80–90	77.25–88.50	
Pre vs F/U		< 0.001	< 0.001	
Bodily pain				
Pre	Median	22	20.50	0.141
	IQR	19–25	17–23.75	
F/U	Median	65.50	61.50	0.170
	IQR	57–79	56–73.75	
Pre vs F/U		< 0.001	< 0.001	
Vitality				
Pre	Median	35	35	0.535
	IQR	25–40	25–40	
F/U	Median	70	65	0.223
	IQR	55–80	60–70	
Pre vs F/U		< 0.001	< 0.001	
Mental health				
Pre	Median	61	62	0.774
	IQR	56–64	57–64.75	
F/U	Median	69	70	0.790
	IQR	65–74	65.25–74	
Pre vs F/U		< 0.001	< 0.001	
General health				
Pre	Median	64.50	63	0.320
	IQR	54–77	53–74	
F/U	Median	69	67	0.351
	IQR	59.75–75.25	58–76	
Pre vs F/U		0.060	0.080	

Pre, preoperative; F/U, follow up; FD, facet degeneration; IQR, interquartile range; VAS, visual analog scale; NDI, neck disability index; SF-36, Short Form-36

**Table 5:** Comparison of clinical outcomes according to preoperative facet degeneration

	Mild-FD group	Severe-FD group	*p*-value
△VAS (neck pain)	4.52 ± 1.68	5.23 ± 1.21	0.010
△VAS (arm pain)	5.47 ± 1.30	5.25 ± 1.06	0.275
△ NDI	17.44 ± 5.13	21.48 ± 4.83	< 0.001
△ Physical functioning	30.59 ± 10.11	30.00 ± 6.68	0.917
△ Social functioning	37.00 ± 8.22	36.38 ± 8.27	0.625
△ Bodily pain	45.04 ± 8.50	43.31 ± 6.72	0.213
△ Vitality	34.12 ± 6.23	35.73 ± 6.19	0.120
△ Mental health	8.45 ± 3.20	8.35 ± 3.62	0.857
△ General health	5.10 ± 14.73	6.21 ± 13.10	0.657

FD, facet degeneration; VAS, visual analog scale; NDI, neck disability index

### Relevant factors for preoperative severe FD

Multivariate logistic regression analysis of the significant parameters on FD demonstrated that age, duration of symptoms, disc height and interfacet distance were independently associated with preoperative FD (age: OR = 1.454, 95% CI 1.213–1.743, *P* < 0.001; duration of symptoms: OR = 1.156, 95% CI 1.023–1.305, *P* = 0.020; disc height: OR = 0.643, 95% CI 0.503–0.821, *P* < 0.001; interfacet distance: OR = 0.383, 95% CI 0.150–0.977, *P* = 0.045; Table 6).

**Table 6:** Multivariate logistic regression analysis of facet degeneration

Parameters	aOR	95%CI	*P*
Age (year)	1.454	1.213–1.743	< 0.001
Duration of symptoms	1.156	1.023–1.305	0.020
Disc height	0.643	0.503–0.821	< 0.001
Interfacet distance	0.383	0.150–0.977	0.045
Pre-VAS (neck pain)	0.850	0.297–2.430	0.761
Pre-NDI	1.143	0.883–1.481	0.310
△VAS (neck pain)	0.855	0.510–1.434	0.552
△ NDI	0.888	0.753–1.045	0.153

aOR, adjusted odds ratio; CI, confidence interval; VAS, visual analog scale; NDI, neck disability index

## Discussion

Our research showed that the age of onset and duration of symptoms in the severe facet degeneration (FD) group were markedly higher than those in the mild FD group, but the disc height and interfacet distance were evidently lower than those in the mild FD group. In addition, compared with the mild FD group, the preoperative VAS (neck pain) score and NDI of the severe FD group were also higher, and the improvement of neck symptoms was better. However, with regard to the patient-oriented SF-36 score, the severity of preoperative FD had no significant effect on the clinical results. Therefore, the degree of preoperative FD was unrelated to the clinical efficacy after anterior cervical discectomy fusion (ACDF). In addition, severe preoperative FD did not significantly impact the changes in cervical sagittal balance before and after surgery. Age, duration of symptoms, disc height and interfacet distance may be relevant factors for FD.

ACDF has been regarded as the “gold standard” for treating cervical degenerative diseases [1], especially cervical spondylotic radiculopathy, which has been confirmed to be related to the maintenance of postoperative sagittal balance [2, 3]. FD has been proven to be remarkably linked to chronic neck pain and degenerative cervical spondylolisthesis [4, 14]. However, whether the degree of FD can be used as a key factor affecting postoperative clinical efficacy remains unknown. Park et al. [7] demonstrated that FD can be analysed using CT images with a 4-grade scoring system, showing clearer information about bone fusion, osteophytes, and joint space. Therefore, we also used the above method as the standard to classify FD. In assessing the severity of FD, we used the mean FD score to divide patients into mild and severe FD groups. There have been many reports on the cervical FD. Although the exact underlying mechanism has not been determined, it has been widely recognized that compared with the lower level of the cervical facet, the higher levels are more prone to degeneration, and the degree of degeneration is relatively higher. In addition, the left side is more prone to degeneration than the right side [7]. Therefore, the asymmetry of the FD may affect the difference in the severity of FD on the left and right sides [15]. Manchikanti et al. considered that the symptoms of chronic neck pain were largely related to FD [5]. However, in the current study, we found that the preoperative severe FD group had more severe neck pain and NDI symptoms than the mild FD group. Previous reports have revealed that intervertebral facet joints can lead to neck pain [16] and that neck symptoms can be effectively relieved by nerve blocking of facet joints, suggesting that facet joints may be a source of pain [17, 18]. Pain nerve fibres densely cover the facet joints [19, 20], and mechanical stimulation can easily trigger the response of the receptors in small facet joints [21, 22]. In addition, the mechanical load on the facet joints may also cause neck pain. FD will cause the sagittal sequence to lose stability, and then it can compensatively generate tensile loads [16]. Furthermore, neck pain may also be induced by the release of inflammatory mediators, nerve compression caused by degenerated osteophytes, and mechanical stimulation [23]. However, no marked differences in VAS, NDI, or SF-36 between the two groups were present at the follow-up period.

In our present study, we evaluated the effects of FD severity on the clinical outcomes of ACDF for the first time. Unfortunately, the results demonstrated that the 2-year ACDF surgical outcomes in radiculopathy patients were not markedly affected by preoperative FD severity. Interestingly, almost all the clinical outcomes will be significantly improved in patients with any preoperative FD severity. In addition, patients with severe FD are more likely to exhibit improved neck-related symptoms than patients with mild FD. A previous report demonstrated that preoperative FD severity was remarkably linked to preoperative neck pain in patients at any age [10]. Although our research did not indicate a correlation between the preoperative severity of FD and preoperative neck pain, we found that age, duration of symptoms, disc height and interfacet distance may be relevant factors for the preoperative severity of FD. The reason may be that FD is a type of degenerative change that is aggravated with age. In this process, the facet joint space is further narrowed and fused, the nucleus pulposus further loses water and shrinks, and the intervertebral disc also narrows. Moreover, the elderly may have greater pain tolerance than the young, which may explain why the severity of FD is related to the duration of symptoms.

The clinical efficacy of cervical spine surgery was evaluated with widely used cervical sagittal parameters, and certain parameters are associated with the scores of postoperative functions [3, 13]. However, the relationship between sagittal parameters and FD has not yet been discussed. Recently, Ling et al. [24] reported the three most important sagittal balance parameters, T1S, cSVA, and SCA, which will be the focus of future research. The results demonstrated that marked changes in postoperative cSVA and SCA, except for T1s, were present in single-segment cervical radiculopathy patients receiving ACDF, but these changes were not linked to the FD degree, indicating that the changes in the overall cervical sagittal parameters may be slightly affected by local FD. Based on the above research and analysis, we consider that ACDF could be an effective surgical treatment for cervical radiculopathy patients with any preoperative FD severity.

However, some limitations warrant further discussion. First, our study included a limited number of cases. Second, the average follow-up time was 26 months, which is too short. A prospective study with a longer follow-up period (e.g., more than 5 years) is necessary to further explore the exact FD effects on the postoperative clinical outcomes of patients with ACDF. Third, we did not perform CT examinations on every follow-up patient, so we could not assess the degree of FD after 2 years or the relationship between FD and clinical scores during the follow-up period. Meanwhile, the proliferation and degeneration of uncinate joint may also affect the postoperative efficacy of ACDF, which may be the focus of future research. However, despite these limitations, our study has value for declaring the association between postoperative clinical outcomes in radiculopathy patients with ACDF and facet degeneration.

## Conclusion

Patients with severe FD tended to experience more severe neck pain before surgery and greater improvement of neck symptoms at the follow-up visit. However, 2-year clinical efficacy and sagittal alignment after ACDF may not be markedly affected by preoperative FD severity. ACDF is considered to be a good choice for patients with radiculopathy, especially for patients with severe FD.

## Data Availability

In attempt to preserve the privacy of patients, clinical data of patients will not be shared; data can be available from the corresponding author upon request.

## Abbreviations

FD: Facet degeneration
ACDF: Anterior Cervical Discectomy and Fusion
SCA: Spino Cranial Angle
T1s: T1-slope
cSVA: C2–C7 Sagittal Vertical Axis
NDI: Neck Disability Index
VAS: Visual Analogue Scale
SF-36: Short Form-36

## References

[1] Liu T, Yang HL, Xu YZ, Qi RF, Guan HQ (2011). ACDF with the PCB cage-plate system versus laminoplasty for multilevel cervical spondylotic myelopathy. J Spinal Disord Tech.

[2] Cabraja M, Abbushi A, Koeppen D, Kroppenstedt S, Woiciechowsky C (2010). Comparison between anterior and posterior decompression with instrumentation for cervical spondylotic myelopathy: sagittal alignment and clinical outcome. Neurosurg Focus.

[3] Tang JA, Scheer JK, Smith JS, Deviren V, Bess S, Hart RA, Lafage V, Shaffrey CI, Schwab F, Ames CP (2012). The impact of standing regional cervical sagittal alignment on outcomes in posterior cervical fusion surgery. Neurosurgery.

[4] Manchikanti L, Boswell MV, Singh V, Pampati V, Damron KS, Beyer CD (2004). Prevalence of facet joint pain in chronic spinal pain of cervical, thoracic, and lumbar regions. BMC Musculoskelet Disord.

[5] Manchikanti L, Singh V, Rivera J, Pampati V (2002). Prevalence of cervical facet joint pain in chronic neck pain. Pain Physician.

[6] Jaumard NV, Welch WC, Winkelstein BA (2011). Spinal facet joint biomechanics and mechanotransduction in normal, injury and degenerative conditions. J Biomech Eng.

[7] Park MS, Lee YB, Moon SH, Lee HM, Kim TH, Oh JB, Riew KD (2014). Facet joint degeneration of the cervical spine: a computed tomographic analysis of 320 patients. Spine.

[8] Aizawa T, Ozawa H, Hoshikawa T, Kusakabe T, Itoi E (2009). Severe facet joint arthrosis caused c7/t1 myelopathy: a case report. Case Rep Med.

[9] Pellengahr C, Pfahler M, Kuhr M, Hohmann D (2000). Influence of facet joint angles and asymmetric disk collapse on degenerative olisthesis of the cervical spine. Orthopedics.

[10] Tamai K, Suzuki A, Yabu A, Takahashi S, Toyoda H, Hoshino M, Terai H, Nakamura H (2019). Preoperative severity of facet joint degeneration does not impact the 2-year clinical outcomes and cervical imbalance following laminoplasty. Spine J.

[11] Tang JA, Scheer JK, Smith JS, Deviren V, Bess S, Hart RA, Lafage V, Shaffrey CI, Schwab F, Ames CP (2015). The impact of standing regional cervical sagittal alignment on outcomes in posterior cervical fusion surgery. Neurosurgery.

[12] Youn MS, Shin JK, Goh TS, Kang SS, Jeon WK, Lee JS (2016). Relationship between cervical sagittal alignment and health-related quality of life in adolescent idiopathic scoliosis. Eur Spine J.

[13] Kandziora F, Pflugmacher R, Schafer J, Born C, Duda G, Haas NP, Mittlmeier T (2001). Biomechanical comparison of cervical spine interbody fusion cages. Spine.

[14] Dean CL, Gabriel JP, Cassinelli EH, Bolesta MJ, Bohlman HH (2009). Degenerative spondylolisthesis of the cervical spine: analysis of 58 patients treated with anterior cervical decompression and fusion. Spine J.

[15] Rong X, Liu Z, Wang B, Chen H, Liu H (2017). The facet orientation of the subaxial cervical spine and the implications for cervical movements and clinical conditions. Spine.

[16] Lee KE, Thinnes JH, Gokhin DS, Winkelstein BA (2004). A novel rodent neck pain model of facet-mediated behavioral hypersensitivity: implications for persistent pain and whiplash injury. J Neurosci Methods.

[17] Leclaire R, Fortin L, Lambert R, Bergeron YM, Rossignol M (2001). Radiofrequency facet joint denervation in the treatment of low back pain: a placebo-controlled clinical trial to assess efficacy. Spine.

[18] Manchikanti L, Singh V, Falco FJ, Cash KM, Fellows B (2008). Cervical medial branch blocks for chronic cervical facet joint pain: a randomized, double-blind, controlled trial with one-year follow-up. Spine.

[19] Cavanaugh JM, Ozaktay AC, Yamashita HT, King AI (1996). Lumbar facet pain: biomechanics, neuroanatomy and neurophysiology. J Biomech.

[20] Giles LG, Harvey AR (1987). Immunohistochemical demonstration of nociceptors in the capsule and synovial folds of human zygapophyseal joints. Br J Rheumatol.

[21] Ashton IK, Ashton BA, Gibson SJ, Polak JM, Jaffray DC, Eisenstein SM (1992). Morphological basis for back pain: the demonstration of nerve fibers and neuropeptides in the lumbar facet joint capsule but not in ligamentum flavum. J Orthopaedic Res.

[22] Yamashita T, Cavanaugh JM (1990). el-Bohy AA, Getchell TV, King AI: Mechanosensitive afferent units in the lumbar facet joint. J Bone Joint Surg Am.

[23] Igarashi A, Kikuchi S, Konno S, Olmarker K (2004). Inflammatory cytokines released from the facet joint tissue in degenerative lumbar spinal disorders. Spine.

[24] Ling FP, Chevillotte T, Leglise A, Thompson W, Bouthors C, Le Huec JC (2018). Which parameters are relevant in sagittal balance analysis of the cervical spine? A literature review. Eur Spine J.

